# A Retrospective Assessment of Four Antigen Assays for the Detection of Invasive Candidiasis Among High-Risk Hospitalized Patients

**DOI:** 10.1007/s11046-017-0238-1

**Published:** 2018-01-22

**Authors:** Barbara Hartl, Iris Zeller, Angelika Manhart, Brigitte Selitsch, Cornelia Lass-Flörl, Birgit Willinger

**Affiliations:** 1 Department of Laboratory Medicine, Division of Clinical Microbiology, Medical University of Vienna, Währinger Gürtel 18-20/5P, A-1090 Vienna, Austria; 2 Present Address: Skånes universitetssjukhus, Getingevägen 4, 222 41 Lund, Sweden; 3 Present Address: Courant Institute of Mathematical Sciences, New York University, 251 Mercer Street, New York, NY 10012 USA; 4 Division of Hygiene and Medical Microbiology, Medical University of Innsbruck, Schöpfstrasse 41, A-6020 Innsbruck, Austria

**Keywords:** Invasive candidiasis, Diagnosis, Antigen, Mannan, BDG, Fungitell

## Abstract

Because of their high mortality rates and non-specific symptoms, invasive *Candida* infections pose a huge diagnostic and therapeutic challenge. In this study, we evaluated the three mannan antigen assays Platelia, Platelia Plus and Serion, and the (1-3)-β-d-glucan assay Fungitell in a group of high-risk (hematological and surgical) patients. Test results of 305 patients hospitalized at the Vienna General Hospital and the University Hospital of Innsbruck were retrospectively analyzed. We assessed the test accuracy by means of descriptive statistics. Nine (2.95%) patients were affected by invasive candidiasis (IC), and 25 (8.2%) patients had a probable/possible infection. The majority of patients (271; 88.9%) showed no signs of infection. The Platelia and Serion mannan assays had a low sensitivity (65% and 52%, respectively), but high specificity (98% for both tests). The newer version of the Platelia assay, the Platelia Plus, had a higher sensitivity (85%) but a lower specificity (89%). The sensitivity of the Fungitell assay was high (100%), while its specificity was low (58%). The positive predictive values were 0.48 for the Platelia and 0.41 for the Serion assay, 0.26 for the Platelia Plus and 0.09 for the Fungitell assay. Our limited, retrospective study suggests the efficacy of mannan assays as screening (Platelia Plus) and confirmatory (Serion) tests, while the Fungitell assay can be used to exclude invasive *Candida* infections.

**Electronic supplementary material:**

The online version of this article (10.1007/s11046-017-0238-1) contains supplementary material, which is available to authorized users.

## Introduction

The early diagnosis of invasive candidiasis (IC) is crucial, but, due to non-specific symptoms and a lack of rapid, reliable diagnostic procedures, very challenging [1–3]. Blood culture is considered the “gold standard” for the detection of IC, even though its sensitivity only lies between 50–75% [4–6]. Different assays based on the detection of fungal cell wall antigens such as mannan and (1–3)-ß-d-glucan (BDG) seem to be promising tools for the fast and sensitive detection of IC [7, 8]. Mannan assays are *Candida*-specific, while BDG assays are able to detect a broad range of fungal species. The Platelia *Candida* Antigen Assay (Bio-Rad Laboratories, Marnes-la-Coquette, France) was evaluated in several studies showing sensitivities and specificities from 29 to 100% and 61 to 100%, respectively [9–12]. According to the European Society of Clinical Microbiology and Infectious Diseases [13], this assay is suitable for the exclusion of IC. The Serion ELISA Antigen *Candida* Assay (Serion GmbH, Würzburg, Germany) has been scarcely discussed in the literature [14, 15] so far. With observed sensitivities and specificities between 47–78% and 70–100%, respectively [9, 16–18], BDG assays are recommended by the European Organization for Research and Treatment of Cancer/Invasive Fungal Infections (EORTC)/Mycoses Study Group (MSG) as suitable tool for the diagnosis of probable invasive fungal infections [19].

We evaluated the diagnostic performance of the Serion assay, the Platelia assay, the newer Platelia *Candida* Antigen Plus (Bio-Rad Laboratories, Marnes-la-Coquette, France) and the most common BDG assay (Fungitell; Associates of Cape Cod, Inc., MA, UA), by retrospectively analyzing test results of a cohort of hematological and surgical patients.

## Materials and Methods

Blood samples of 305 hospitalized patients were collected at the Vienna General Hospital and the University Hospital of Innsbruck and tested with Platelia, Serion and Fungitell. In addition, 289 out of these patients were also tested with the Platelia Plus assay. The study included surgical intensive care patients as well as patients with leukemia and neutropenia of at least 14 days. Based on information from medical records and histopathological, radiological and microbiological findings, the patients were classified into three risk groups (proven IC, possible/probable IC and no IC) according to the EORTC/MSG criteria. The limited access to some medical findings did not allow further differentiation between possible and probable IC. As our study was designed as retrospective analysis, the test results did not influence treatment decisions and thus did not affect the therapeutic outcomes.

### Statistics

Test accuracy was assessed using descriptive statistics and calculating sensitivity, specificity, positive predictive value (PPV) and NPV. As multiple testing was not performed in all patients, the assays results were evaluated per sample and not per patient.

## Results

Two hundred and five (67.2%) out of 305 tested patients had been hospitalized at the University Hospital of Vienna, 100 (32.7%) at the University Hospital of Innsbruck. In total, 189 (62.0%) patients were men and 116 (38.0%) women. The patients’ ages ranged from 7 to 94 years (median 59 years).

**Fig. 1:**
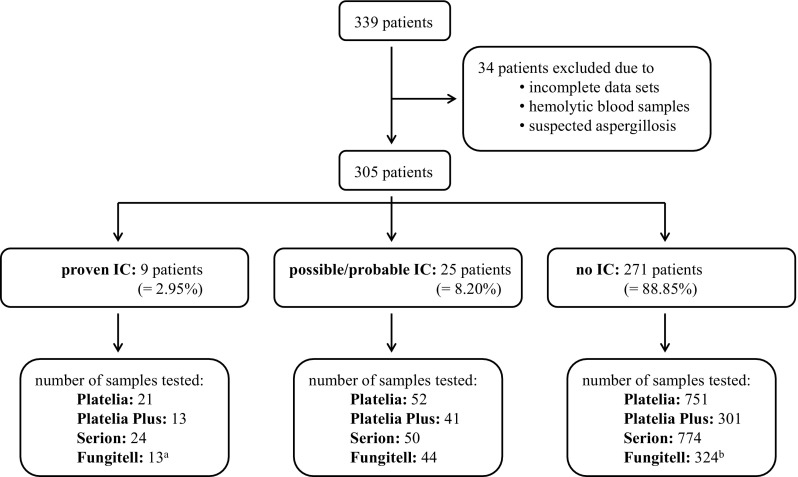
Number of samples tested with Platelia, Platelia Plus, Serion and Fungitell among the three different patient groups. ^a^ Only samples from 6 of the patients with proven IC were tested with Fungitell in close proximity to the time of IC diagnosis. ^b^ Samples from the 3 other patients that were only tested outside of the required time frame of ± 7 days were added to the no IC group (274 patients)

Nine (3%) out of 305 patients showed evidence of *Candida* species in samples from sterile sites at least once in close temporal relation (± 7 days) to the date of antigen testing and were therefore regarded as patients with proven IC. According to the EORTC/MSG criteria and in the context of other diagnostic findings and clinical symptoms, 25 (8.2%) patients were assigned to the group of possible/probable IC, whereas 271 (88.9%) patients showed no signs of IC. All in all, 824 blood samples were tested with the Platelia, 355 with the Platelia Plus, 848 with the Serion and 381 with the Fungitell assay (Fig. 1). Supplemental Table 1 summarizes inconclusive test outcomes.

### Patients with Proven IC

The majority of patients (7/9 = 77.8%) with proven IC were surgical patients, one patient had leukemia, while details regarding the underlying disease of another patient were not accessible for the analysis. As shown in Table 1, 5 out of 9 (55.6%) infected patients showed positive results with the Platelia and the Platelia Plus assay, while samples from 4 (44.5%) patients were positive upon testing with the Serion assay. The Fungitell assay was only conducted on samples from 6 patients and yielded a positive result in all cases, even though 1 out of 2 test results for a patient with confirmed *C. lusitaniae* sepsis was inconclusive. IC was successfully detected in 4 of the 9 patients by all four antigen assays. When the analysis was based on individual samples rather than patients, the Platelia and Serion assays showed detection rates of 61.90% for the Platelia and 50% for the Serion assay. The Platelia Plus (84.62%) and the Fungitell (92.31%) exhibited higher detection rates (Table 1). Patients were infected with *C. albicans* (5/9 = 55.5%), *C. glabrata* (2/9 = 22.2%), *C. parapsilosis* (1/9 = 11.1%) and *C. lusitaniae* (1/9 = 11.1%). *C. albicans* was detected in 7/13 (53.9%) samples with the Platelia, in 3/5 (60%) with the Platelia Plus and 7/15 (46.7%) samples with the Serion assay, while detection rates for *C. glabrata* were 4/5 (80%) with the Platelia, 6/6 (100%) with the Platelia Plus and 3/6 (50%) with the Serion antigen assay. The Fungitell detected *C. albicans* and *C. glabrata* in 100% of the samples from patients with proven IC. *C. lusitaniae* was detected in 2/2 samples with the Platelia, Platelia Plus and the Serion assay, while Fungitell gave an inconclusive result in 1 out of 2 tests. In our study, only 1 patient was affected by *C. parapsilosis* and remained undetected by both mannan assays used; the BDG assay was not performed on the sample submitted from this patient within the ± 7 day window (Table 2).

**Table 1:** Patients with proven IC

Patient	Diagnosis	Blood culture	Other results	Antifungal treatment	positive results/samples tested ± 7 days of diagnosis			
					Platelia 13/21 (61.90%)	Platelia Plus 11/13 (84.62%)	Serion 12/24 (50%)	Fungitell 12/13 (92.31%)
1	Candidemia	*C. albicans*	–	Yes	0/2	–	0/3	–
2	Candidemia	*C. parapsilosis*	–	Yes	0/1	–	0/1	–
3	Deep organ candidiasis	–	Pleural aspirate: *C. glabrata*	Yes	3/3	3/3	3/3	3/3
4	Candidemia	*C. albicans*	–	Yes	0/1		0/1	–
5	Candidemia	*C. albicans*	–	Yes	3/3	1/1	3/3	1/1
6	Candidemia	*C. albicans*	PCR: *C. albicans*	Yes	4/6	2/2	4/6	2/2
7	Endocarditis	–	Histology + culture: *C. albicans*	Unclear	0/1^b^	0/2^b^	0/2^a^	2/2
8	Candidemia	–	PCR: *C. glabrata*	Yes	1/2^a,b^	3/3^b^	0/3	3/3
9	Candidemia	*C. lusitaniae*	BAL: *C. lusitaniae*	Yes	2/2	2/2	2/2	1/2^a^

^a^1 sample yielded in an inconclusive result

^b^1 serum sample obtained was only tested with the Platelia Plus assay

**Table 2:** Detection rates for different *Candida* species in patients with proven IC

	Positive results/samples tested ± 7 days of diagnosis			
	*C. albicans*	*C. glabrata*	*C. parapsilosis*	*C. lusitaniae*
Platelia (*n* = 21)	7/13 (53.85%)	4/5 (80%)	0/1 (0%)	2/2 (100%)
Platelia Plus (*n* = 13)	3/5 (60%)	6/6 (100%)	–	2/2 (100%)
Serion (*n* = 24)	7/15 (46.67%)	3/6 (50%)	0/1 (0%)	2/2 (100%)
Fungitell (*n* = 13)	5/5 (100%)	6/6 (100%)	–	1/2 (50%)

### Sensitivity, Specificity, PPV, NPV

Samples that yielded in inconclusive results were excluded from further analysis. We used 2 different approaches to calculate sensitivity, specificity, PPV and NPV. First, we only included samples from patients with proven IC that were obtained ± 7 days of the definite diagnosis as well as samples from patients without IC in our analysis. Additionally, we calculated these parameters when samples from patients with possible/probable IC were included in the group of proven IC patients. The false-positive as well as the false-negative rates were quite similar among the Platelia and Serion assays, whereas no false-negative results but a high rate of false positives were observed upon Fungitell testing in patients with proven IC. Also the Platelia Plus assay showed a considerably higher rate of false-positive results (Table 3).

**Table 3:** Test characteristics of the four antigen assays

	Platelia		Platelia plus		Serion		Fungitell	
	Proven IC	Proven, probable or possible IC	Proven IC	Proven, probable or possible IC	Proven IC	Proven, probable or possible IC	Proven IC	Proven, probable or possible IC
True positives	13	29	11	20	12	26	12	34
True negatives	709	709	259	259	710	710	170	170
False negatives (false-negative rate)	7 (35%)	41 (58.5%)	2 (15.4%)	31 (60.8%)	11 (47.8%)	44 (62.9%)	0 (0%)	17 (33.3%)
False positives (false-positive rate)	14 (1.9%)	14 (1.9%)	31 (10.7%)	31 (10.7%)	17 (2.3%)	17 (2.3%)	123 (42%)	123 (42%)
Total	743	793	303	341	750	797	305	344
Sensitivity	0.65	0.41	0.85	0.39	0.52	0.37	1	0.67
Specificity	0.98	0.98	0.89	0.89	0.98	0.98	0.58	0.58
PPV	0.48	0.67	0.26	0.39	0.41	0.60	0.09	0.22
NPV	0.99	0.95	0.99	0.89	0.98	0.94	1	0.91

### Colonization

Apart from the 9 infected patients, 31 (10.4%) of the remaining 296 patients showed signs of colonization at the time of antigen testing. Among these colonized patients, 7 (22.6%) gave false-positive results with the Platelia assay, 5 (16.1%) with the Serion assay and 18 (58.1%) with the Fungitell. The numbers of false positives among the group of non-colonized patients were 3 (1.1%) with the Platelia assay, 5 (1.9%) with the Serion assay and 72 (27.2%) with the Fungitell.

## Discussion

The major benefits of antigen testing are the short time to result and its cost-effectiveness. However, inconsistent observations regarding the test accuracies were reported in previous studies. This might be due to the heterogeneity of the available studies and the low prevalence of IC, since small numbers of positive samples make it difficult to draw conclusions of statistical significance. Among intensive care patients, for example, the prevalence was reported to only lie between 0.5 and 1% [20]. In our study, 9 out of 305 patients were classified as patients with “proven IC,” which corresponds to a prevalence of 2.95% (Fig. 1).

For the Platelia assay, our study detected a sensitivity of 65%, (41% when possible/probable cases were included) and a specificity of 98% (Table 3). In a meta-analysis conducted by Mikulska et al. [11], an overall sensitivity of 58% and specificity of 93% were reported. We tested samples from 289 out of 305 patients also with the newer version of the Platelia assay, the Platelia *Candida* Antigen Plus assay. This assay—which today is the only available Platelia *Candida* Antigen assay—was advertized to have a lower limit of detection compared to its predecessor. Lunel et al. [21] compared both tests and observed a modest increase in sensitivity, while the specificity was reduced by half due to a high number of false-positive results in patients with superficial candidiasis. We were able to detect an increase in sensitivity to 85%, while the decrease in specificity (89%) was less prominent. The number of false-positive tests increased from 14 to 31 with Platelia Plus, whereas the number of false-negative results decreased from 7 to 2.

The Serion assay showed a sensitivity of 52% (37% if possible/probable cases were included) and a specificity of 98% in our study. Lunel et al. [15] observed a sensitivity of 70% and specificity of 80% in patients with neutropenia lasting for less than 15 days, while in patients with prolonged neutropenia the sensitivity decreased to 46% and the specificity increased to 100%. A sensitivity of 77% and specificity of 51% was reported by Chumpitazi et al. [14].

Both mannan tests gave false-negative results for patient 2, a patient diagnosed with *C. parapsilosis* candidemia. This might be due to the fact that the antibodies used only have weak reactivity for the antigens from *C. parapsilosis* and *C. krusei* as was shown by Rimek et al. [22] for the Platelia assay. Sendid et al. [23] also observed poor detection of these species, and Yera at al. [24] reported detection rates 44% for C. *krusei* and *C. parapsilosis* with the Platelia assay.

Since time to detection is a very important factor in the diagnosis of invasive infections, we examined the applicability of antigen tests for the early detection of IC. Several studies suggest that antigen testing is superior to culture in terms of time to diagnosis [11] 26]. Even though only results obtained ± 7 days of the definite (cultural or molecular) diagnosis of IC were included in the analysis of test quality parameters, results obtained outside of this 7 day window were reviewed. In 5 out of the 9 patients with proven IC, antigen assays gave positive results several days before culture positivity. All mannan assays as well as the Fungitell assay gave positive results 21 days before the culture results were available for patient 3 (*C. glabrata* was identified in the patient’s pleural aspirate). However, at least 2 of these patients (patients 3 and 5) were also colonized by *Candida*, and it is not possible to evaluate whether the antigen assays detected the colonization or the infection.

The impact of fungal colonization on false-positive results and the low specificity remains unclear [10, 25]. Nichterlein et al. [26] performed an animal study in which mice were infected with *C. albicans* systemically or gastrointestinally. The ability to differentiate between disseminated infection and mere colonization was examined for commercial mannan and BDG assays. Mice without detectable dissemination remained negative in the BDG assay but showed positive or intermediate results in the mannan antigen assay. Due to a lack of data in our study, only a part of the patients colonized at the time of testing could be identified. The number of false-positive results in the group of 31 colonized patients was much higher than in the group of 265 non-colonized patients regardless of the type of assay used.

In summary, despite the large number of patients in our study the statistical power is limited due to the low prevalence of IC. Nevertheless, based on the high specificity and low number of false positives we consider the mannan tests qualified for the confirmation of IC. Due to the higher specificity, the Serion assay might be the better confirmatory test, while the Platelia Plus could be used as a screening test. Its high sensitivity and high NPV make the Fungitell assay a valuable tool for the exclusion of IC, as has already been recommended [13]. In accordance with Poissy et al. [25], we recommend the combined use of mannan and BDG assays. Furthermore, these methods should only be used in combination with other diagnostic methods and interpreted in the context of clinical symptoms. Future studies could include other means of detecting invasive *Candida* infections, such as a *Candida albicans* germ-tube antibody assay [27, 28], and should focus on the analysis of serial antigen testing instead of single antigen test results. Larger, prospective studies are needed in order to develop reliable guidelines for an efficient use of antigen assays in practice.

## Electronic supplementary material

Below is the link to the electronic supplementary material.

Supplementary material 1 (DOCX 48 kb)
