# Supplementary material for: A Retrospective Assessment of Four Antigen Assays for the Detection of Invasive Candidiasis Among High-Risk Hospitalized Patients
Source: Mycopathologia. 2018 Jan 22;183(3):513–9. doi: 10.1007/s11046-017-0238-1 (PMC5958149; doi:10.1007/s11046-017-0238-1)
Supplement: Supplementary file 1 — Supplementary material 1 (DOCX 48 kb) [file 11046_2017_238_MOESM1_ESM.docx]

**Supplemental Table 1 – Samples yielding inconclusive test results**

|  | | **Inconclusive Test Results** |
| --- | --- | --- |
| **Platelia**  **(n=824)** | **Proven IC** | 1 |
|  | **Possible/Probable IC** | 2 |
|  | **No IC** | 28 |
| **Platelia Plus**  **(n=355)** | **Proven IC** | 0 |
|  | **Possible/Probable IC** | 3 |
|  | **No IC** | 11 |
| **Serion**  **(n=848)** | **Proven IC** | 1 |
|  | **Possible/Probable IC** | 3 |
|  | **No IC** | 47 |
| **Fungitell**  **(n=381)** | **Proven IC** | 1 |
|  | **Possible/Probable IC** | 5 |
|  | **No IC** | 31 |
